# Assessment of health-related quality of life in antiviral-treated Taiwanese chronic hepatitis C patients using SF-36 and CLDQ

**DOI:** 10.1186/1477-7525-12-97

**Published:** 2014-06-18

**Authors:** Shu-Chuan Chang, Sheng-Shun Yang, Chiu-Chun Chang, Chun-Che Lin, Yueh-Chin Chung, Tsai-Chung Li

**Affiliations:** 1 Department of Nursing, Central Taiwan University of Science and Technology, Taichung, Taiwan; 2 Department of Public Health, China Medical University, Taichung, Taiwan; 3 Division of Gastroenterology & Hepatology, Department of Internal Medicine, Taichung Veterans General Hospital, Taichung, Taiwan; 4 School of Medicine, National Yang-Ming Medical University, Taipei, Taiwan; 5 Institute of Medicine, Chung Shan Medical University, Taichung, Taiwan; 6 Division of Gastroenterology & Hepatology, Chung Shan Medical University Hospital, Taichung, Taiwan; 7 Graduate Institute of Biostatistics, College of Management, China Medical University, Taichung, Taiwan; 8 Department of Healthcare Administration, College of Health Science, Asia University, Taichung, Taiwan

**Keywords:** Chronic hepatitis C, Antiviral treatment, Health-related quality of life

## Abstract

**Background:**

Interferon (IFN) therapy can cause significant side effects in chronic hepatitis C (CHC) patients; however, the health-related quality of life (HRQOL) of antiviral-treated CHC patients has not been established in Taiwan. This study evaluated domains and the degree to which antiviral treatment affects the HRQOL in CHC patients and identifies factors associated with variations between patients.

**Methods:**

Health-related quality of life (HRQOL) was assessed using the Short Form-36 (SF-36) and the Chronic Liver Disease Questionnaire (CLDQ) in 108 antiviral-treated CHC patients. Eight scales and two summary scales of the SF-36 were compared with 256 age- and gender-matched population norms and 64 age- and gender-matched CHC patients without antiviral therapy. Descriptive statistic measures, one-way ANOVA, and regression analysis were used for data analysis.

**Results:**

(1) CHC patients receiving antiviral treatment displayed significantly lower scores in six scales, the Physical Component Summary (PCS), and the Mental Component Summary (MCS) of the SF-36, when compared to the population norms and patients without antiviral therapy (*p* < 0.05). (2) The mean CLDQ score of antiviral-treated patients was lower than that of patients without antiviral therapy, including subscales of ‘fatigue’, ‘systemic symptoms’, and ‘role emotion’. (3) All SF-36 subscales significantly correlated with all CLDQ subscales, with the greatest correlation coefficients shown between fatigue and vitality and mental health of SF-36. (4) Antiviral therapy had a greater negative impact on females in the CLDQ, on all patients during treatment weeks 9–16 in the PCS and on patients with a monthly income of less than NT$10,000 in the CLDQ, PCS, and MCS.

**Conclusions:**

This study highlighted impairments in the quality of life of chronic hepatitis C patients treated with IFN-based therapy. The significant factors associated with HRQOL include gender, income, and treatment duration. The results of this study might provide nurses with a comprehensive understanding of HRQOL and its determining factors in antiviral-treated CHC patients. The findings can serve as a useful reference for nursing personnel in developing instructions for upgrading the care of CHC patients.

## Introduction

Chronic hepatitis C (CHC) is one of the major causes of liver cirrhosis and hepatocellular carcinoma (HCC). Previous research indicated the prevalence of anti-hepatitis C virus (HCV) antibody positivity in Taiwan was 4.4%, with the total number of HCV carriers in the general population reaching approximately 420,000 [1]. It is estimated that 85% of HCV-infected individuals will progress to CHC [2]. Over time, approximately 35% of patients with CHC will progress to cirrhosis and 20% of patients will develop HCC [3,4]_._

Interferon (IFN) plus ribavirin therapy is the current standard of care for antiviral treatment of CHC [5,6]; however, this treatment causes adverse events, including fatigue, myalgia, influenza-like symptoms, alterations in mood, the inability to concentrate, and changes in libido. These events may have a negative effect on a patient’s vitality, social interaction, and ability to perform work and other activities, and may force a discontinuation of therapy [7,8]. Although qualitative and quantitative study methods have shown that IFN-based treatment for CHC patients has a negative impact on health-related quality of life (HRQOL) [9-11], studies of the impact of IFN-based treatment on HRQOL in CHC patients are limited.

Generally, it is recommended that both generic and disease-specific measures be used in HRQOL surveys or trials. Generic measures provide comparisons between general populations and patients with chronic conditions, whereas disease-specific measures assess disease-specific symptoms and are more sensitive to changes, including small yet clinically significant changes. Disease-specific measures can capture patients’ experiences throughout the course of a disease and its treatment. The Short Form 36 Questionnaire (SF-36) is one tool commonly used as a generic measure to assess HRQOL. It has gained popularity as a means of evaluating outcomes in a wide variety of patient groups and surveys. HRQOL measures specifically developed in previous decades for chronic liver disease include the Chronic Liver Disease Questionnaire (CLDQ) and the Hepatitis Quality of Life Questionnaire (HQLQ). The CLDQ is the most commonly used instrument and has the advantage of being a short and feasible questionnaire [12]. On the contrary, the HQLQ is considered too lengthy and lacking validity [13,14].

Studies of HRQOL of CHC patients undergoing treatment have shown that antiviral treatment has an enormous impact on HRQOL compared with untreated patients [15-20]. Reports indicate that the HRQOL of untreated CHC patients either resembles the general population [21] or is lower than that of healthy individuals [20,22,23]. Two previous studies addressed the HRQOL in CHC patients undergoing treatment in countries with a high HCV prevalence such as Taiwan. One study used disease-specific scales of the Hepatitis Quality of Life Questionnaire (HQLQ) [24], while the other applied the Taiwanese version of the WHOOQOL-BREF [25]. Neither study evaluated the HRQOL in CHC patients undergoing treatment using disease-specific and generic measures simultaneously. The present study used the SF-36 to determine the domains in which antiviral treatment affects CHC patients and the degree to which patients are affected compared with Taiwanese population norms on 10 scales of the SF-36. The study also aimed to identify determinants of HRQOL as measured by SF-36 and CLDQ.

## Materials and methods

This study used a cross-sectional design. Questionnaires were used to interview antiviral-treated CHC patients at outpatient clinics in one medical center in central Taiwan. Patients were diagnosed with CHC if they had both HCV antibody and HCV RNA positivity for more than six months, elevated serum alanine aminotransferase (ALT) levels, and diagnostic confirmation by experienced hepatologists. One hundred and eight participants were currently undergoing pegylated IFN plus ribavirin therapy, while 84 participants were recruited for the non-treated groups. To compare the HRQOL, 64 antiviral-treated CHC patients were frequency-matched based on their age and gender with 64 CHC patients without antiviral therapy. Subjects were interviewed before filling out questionnaires with the interviewer’s assistance during outpatient clinic visits from July 1, 2010 to December 31, 2011. Patients’ baseline characteristics, including age, gender, marital status, educational level, current employment, monthly income, on-visit ALT levels, and treatment duration were collected. The investigators administered two questionnaires: the generic Short Form 36 (SF-36) and the CLDQ. The SF-36 consists of thirty-six items divided into eight scales that can be combined into two summary scores: the mental component summary (MCS) and the physical component summary (PCS). The SF-36 scales are: 1) physical functioning (PF), 2) role physical (RP), 3) bodily pain (BP) 4), general health (GH), 5) vitality (VT), 6) social functioning (SF), 7) role emotion (RE), and 8) mental health (MH). Scores for these SF-36 scales range between 0 and 100, with higher scores indicating a better HRQOL. The tool is well-established as a valid measurement and is widely considered to yield adequate data quality. The scales are derived from the standard SF-36 scoring algorithms [26].

The Chinese (Taiwan) SF-36 Health Survey has been translated, validated, and normalized for the general Chinese population in Taiwan. Previous reports on the norms and internal consistency of the Taiwanese version of the SF-36 indicate that the reliability coefficient of internal consistency for the total scale was 0.86 [27,28]. A total of 2,165 subjects from the normal population were randomly selected to take part in the SF-36 in this study. Norms were selected using a four-stage sampling design from a target population of residents in one city and four counties of Taiwan in 1994 [29], from the same residential area as antiviral-treated CHC patients. Individuals aged less than 27 years and more than 77 years were excluded for the sake of comparable distributions of age and gender between the population norms, and 256 of the population norms were frequency-matched by age and gender.

The CLDQ, developed by Younossi et al. [12], has been used in most recent studies of HRQOL in patients with CLD. The questionnaire contains 29 items divided into domains: abdominal symptoms (AB), activity (AC), emotional function (EM), fatigue (FA), systemic symptoms (SY), and worry (WO). Overall CLDQ scores calculated for each domain range from 1 (most impaired) to 7, with higher scores indicating a minimum frequency of symptoms and hence a better HRQOL. The total score is calculated as the average score of the 29 items. The original questionnaire has good test-retest reliability (r = 0.90) and cross-sectional validity [12]. The Chinese version in Taiwan was translated using the forward and backward translation method in 2011 [30]. The resulting instrument was rated by content validity index (CVI) for validity and Cronbach’s alpha and test-retest reliability for reliability. The CVI value was 0.86 for each item and the reliability coefficient of two tests for the total scale was above 0.92 in this study. IRB approval was obtained from the investigators’ affiliated universities, as well as the hospital where patients were recruited. All participants provided written informed consent after the study purpose, procedures, and the right to withdraw had been explained in detail.

## Data analysis

Descriptive analysis was applied to all antiviral-treated patients (n = 108) for baseline characteristics, the SF-36 questionnaire, and the CLDQ. One-way ANOVA was used to estimate the differences between the 64 antiviral-treated CHC patients, the 64 patients not undergoing antiviral therapy, and the 256 Taiwanese population norms, including the 8 domains and 2 component summary scales of the SF-36. An independent *t*-test was used to estimate the differences between antiviral-treated CHC patients and CHC patients without antiviral therapy, including the six domains of the CLDQ. Baseline characteristics were chosen for independent variables to predict factors on PCS, MCS, and the CLDQ via multiple linear regression. Demographic items included age, gender, marital status, education, current employment, monthly income, participants’ baseline clinical characteristics on interview (including serum ALT levels), and treatment duration obtained from medical records. Data analysis was conducted in SPSS for Windows 12.0 (SPSS Inc., Chicago, IL), with two-sided *p* < 0.05 set as statistical significance.

## Results

### Baseline characteristics of 108 antiviral-treated CHC patients

Of the 108 enrolled patients, 59 (54.6%) were male. Age ranged from 27 to 77 years, with an average age of 55.2 years. Over half of the patients (57.4%) had an education level above junior high school, while 82 patients (75.9%) were married and lived with their families. Most patients (56.5%) were currently employed, though only 35.2% had a monthly income more than NT$30,000. The mean ALT level was 50.52 U/L (SD = 50.01). Mean treatment duration at patient interviews was 11.19 weeks (SD = 7.78), while the treatment duration of 59 patients (54.6%) ranged from 1 to 8 weeks (Table 1).

**Table 1:** Demographic and clinical characteristics of patients (n = 108)

**Items**	**Number**	**Percentage (%)**
Gender		
Male	59	54.6
Female	49	45.4
Marital status		
Married	82	75.9
Single, divorced, or widowed	26	24.1
Educational level		
Elementary school and below	46	42.6
Junior and Senior high school	42	38.9
College and above	20	18.5
Occupation		
Employed	61	56.5
Unemployed	47	43.4
Monthly Income		
≤NT$10,000	48	44.4
NT$10,001–30,000	22	20.4
>NT$30,000	28	35.2
Treatment duration on interview		
≤ 8 weeks	59	54.6
9–16 weeks	27	25.0
≥17 weeks	22	20.4
ALT level*		
Mean (SD)	50.52 (50.01)	

*ALT = alanine transaminase; SD = standard deviation.

### Quality of life in antiviral-treated CHC patients compared with population norms and patients without antiviral therapy

In 108 recruits, the average score of ‘role physical’ was lowest, with a score of 37.50 (SD = 44.36), while ‘physical functioning’ was highest, with a score of 78.33 (SD = 20.29). Three scales and two component summary scales scored below 50: role physical, vitality (49.07, SD = 22.87), role emotion (45.35, SD = 47.03), PCS (41.65, SD = 12.42), and MCS (46.02, SD = 12.00). Compared with the population norms, the mean differences in the six SF-36 domains, with the exception of PF and BP, were significantly lower. The maximal reduction in scores for patients was approximately 37.0 points below predicted scores for norms on the role physical scale. CHC patients undergoing antiviral treatment also scored significantly lower on the two component summary scales compared with general norm populations (*p* < 0.05). Compared with CHC patients without antiviral therapy, significantly lower scores on five scales, namely, RP, VT, RE, and MH of SF-36, and PCS, were observed in antiviral-treated CHC patients (*p* < 0.01), as shown in Table 2.

**Table 2:** Scores of SF-36 between antiviral-treated CHC patients, Taiwanese general populations, and patients without antiviral therapy

**SF-36 domains & CLDQ**	**General populations (N = 256,A) mean (SD)**	**CHC without antiviral-treated patients (N = 64,B) Mean (SD)**	**Antiviral-treated CHC patients (N = 64,C) Mean (SD)**	**F¸ (post-hoc multiple comparison) t-test**
Physical functioning	82.79 (22.16)	71.95 (29.33)	75.86 (21.85)	6.57** (A > B)
Role physical	72.95 (41.34)	66.07 (45.36)	35.94 (44.29)	19.19** (A > C&B > C)
Bodily pain	74.26 (17.97)	80.63 (25.04)	73.30 (22.84)	2.88
General health	64.78 (19.96)	41.84 (28.57)	51.78 (20.99)	32.35** (A > C > B)
Vitality	65.33 (16.80)	60.94 (25.91)	50.00 (25.09)	15.01**(A,B > C)
Social functioning	83.35 (15.98)	81.53 (22.49)	75.23 (24.39)	4.78**(A > C)
Role emotional	79.43 (37.72)	78.11 (37.70)	43.20 (46.66)	22.32** (A,B > C)
Mental health	70.91 (14.77)	71.88 (18.43)	61.00 (20.51)	10.10** (A,B > C)
PCS	48.39 (8.52)	52.97 (17.20)	40.78 (12.50)	20.06** (B > A > C)
MCS	49.73 (8.19)	49.73 (12.00)	46.34 (12.51)	3.23* (A > C)
Abdominal symptom		6.06 (1.30)	5.75(1.33)	1.31
Fatigue		5.38(1.56)	4.78(1.38)	2.31*
Systemic symptoms		5.71(1.09)	4.98(1.06)	3.88**
Activity		5.12(1.51)	5.21(1.52)	1.01
Emotional function		5.78(1.16)	5.02(1.42)	3.30**
Worry		6.10(1.33)	5.95(1.16)	0.71

*p < 0.05, **p < 0.01; Age and gender were matched; SD: standard deviation; post-hoc multiple comparison by Scheffe’ method.

The mean CLDQ score of 108 patients was 5.27 (SD = 0.96). The two lowest scores out of the six 3CLDQ subscales were ‘fatigue’ (average 4.66, SD = 1.34) and ‘systemic symptoms’ (average 5.01, SD = 1.04), while the highest was ‘worry’ (average 5.97, SD = 1.12), indicating that most patients had serious symptoms of fatigue, as well as systemic symptoms such as itchy skin, dry mouth, muscle cramps, and dyspnea (Table 3). Compared to patients without antiviral therapy, significantly lower scores on three scales, specifically, fatigue, systemic symptoms, and emotional function, of the CLDQ, were noted in antiviral-treated CHC patients (*p* < 0.05), as shown in Table 2.

**Table 3:** Pearson’s correlation coefficients between CLDQ subscales and SF-36 domains (n = 108)

**CLDQ**	**Mean (SD)**			**SF-36**							
**Items**		**PF**	**RP**	**BP**	**GH**	**VT**	**SF**	**RE**	**MH**	**PCS**	**MCS**
Abdominal symptoms	5.72 (1.30)	_0.16_	0.21*	0.39**	0.32**	0.26**	0.37**	0.31**	0.39**	0.21*	0.41**
Fatigue	4.66 (1.34)	0.46**	0.55**	0.57**	0.54**	0.77**	0.63**	0.55**	0.49**	0.52**	0.70**
Systemic symptoms	5.01 (1.04)	0.43**	0.47**	0.53**	0.36**	0.53**	0.56**	0.49**	0.44**	0.49**	0.52**
Activity	5.17 (1.56)	0.43**	0.47**	0.39**	0.33**	0.45**	0.45**	0.38**	0.31**	0.48**	0.38**
Emotional function	5.08 (1.36)	0.47**	0.51**	0.45**	0.53**	0.58**	0.59**	0.59**	0.74**	0.42**	0.75**
Worry	5.97 (1.12)	0.38**	0.30**	0.40**	0.41**	0.37**	0.44**	0.36**	0.48**	0.35**	0.48**

*p < 0.05; ** *p*< 0.01.

### Correlations between scores of SF-36 instruments and the CLDQ for 108 antiviral-treated CHC patients

All SF-36 subscales correlated significantly with all scores of CLDQ subscales (r > 0.20), ranging from weak to moderate, with the exception of the physical functioning scale of the SF-36 having no significant correlation with the abdominal symptom domain of the CLDQ (Table 3). The strongest association was between the fatigue subscales of the CLDQ and all SF-36 scales, followed by the associations between scales of systemic symptoms and emotional function.

### Predictors of total HQLQ for 108 antiviral-treated CHC patients

Multiple linear regression analysis predicted the PCS, MCS, and CLDQ scores. Table 4 shows beta coefficients obtained from each independent variable and the *R*2 values obtained from each regression model, which measures the proportion of variance in the dependent variable accounted for in the regression model. Significant predictors of PCS include treatment duration (9–16 weeks; *β* = -9.38) and monthly income (greater than NT$30,000; *β* = 8.47). Results for the PCS showed a significant positive correlation with monthly income and a negative correlation with treatment duration. Regression models explained 28% of total variation in the PCS. The standardized regression coefficient showed that the monthly income and 9–16 weeks treatment duration had the greatest impact on PCS scores.

**Table 4:** Multivariate linear regression** for predictive factors on PCS, MCS, and CLDQ (n = 108)

**Variables entered**	**PCS**			**MCS**			**CLDQ**		
	***β*** **(SE)**	***P***	**b**	***β*** **(SE)**	***P***	**b**	***β*** **(SE)**	***P***	**b**
Treatment duration (<8 weeks as reference)									
9–16 weeks	-9.38(2.69)	0.00	-0.33	0.66(2.81)	0.82	0.02	-0.32(0.22)	0.16	-0.15
>17 weeks	-3.83(2.88)	0.19	-0.13	1.56(3.00)	0.60	0.05	0.09(0.24)	0.72	0.04
Monthly income (≤NT$10,000 as reference)									
>NT$30,000	8.47(3.54)	0.02	0.33	8.79(3.69)	0.02	0.35	0.68(0.29)	0.02	0.34
NT$10,001 ~ 30000	5.69(3.39)	0.10	0.19	7.13(3.54)	0.05	0.24	0.37(0.28)	0.19	0.16
Gender (female as reference)	4.39(2.44)	0.08	0.18	4.80(2.54)	0.06	0.20	0.43(0.20)	0.04	0.23
R^2^	0.28			0.16			0.16		

*β =* regression coefficient, b = standardized regression coefficient, SE = standard error.

**Adjusted for age, gender, marital status, education, current employment, and serum ALT level.

The significant predictor of the MCS was a monthly income greater than NT$30,000 (*β* = 0.88). Results of the MCS showed a significant positive correlation with the patient’s monthly income. Regression models explained 16% of the total variation in the MCS. Standardized regression coefficient showed that monthly income had the greatest impact on MCS scores.

Significant predictors of the CLDQ included gender (male, *β* = 0.43) and monthly income greater than NT$30,000 (*β* = 0.68). Regression models explained 16% of the total variation in the CLDQ. The standardized regression coefficient showed that the monthly income and gender had the greatest impact on CLDQ scores.

## Discussion

This study rated the effect of IFN-based treatment for CHC patients on subjective function and well-being. The key results indicated that antiviral-treated CHC patients experienced a marked decrease in scores on 6 scales, as well as the PCS and MCS of the SF-36, ranging from 3.4 (MCS) to 37.0 (RP) points below the scores of the Taiwanese population norms. In 4 scales, as well as the PCS of the SF-36, ranged from 3.4 (MCS) to 34.9 (RE) points below the scores of patients without antiviral therapy. Assuming clinically meaningful differences of 3–5 points [31], the differences on all scales for CHC patients with IFN-based treatment after adjusting for age and gender were substantial, especially for scores on the RP and RE scales. The strongest association between questionnaires was between the fatigue subscale of the CLDQ and the VT and SF scales of the SF-36. In addition, we identified female gender, treatment duration at 9–16 weeks, and monthly income as significant factors affecting the HRQOL.

It is well-known that the majority of HCV-infected patients are asymptomatic seropositive during its natural course; however, HCV itself may decrease the HRQOL in the absence of advanced liver disease [32-34]. A systematic review by Spiegel et al. identified 15 studies that compared HRQOL in compensated HCV-seropositive patients versus healthy controls without HCV, revealing decreased weighted mean SF-36 scores including 7.0 (PF), 15.8 (RP), 12.8 in MCS, and 9.11 in PCS [34]. More recent studies reported that CHC patients’ HRQOL was worse than non-CHC patients, with CHC patients showing significantly lower SF-36 scores in six domains, especially in the VT, GH, and RP scales [22,23,35]. In the present study, CHC patients undergoing antiviral therapy showed significantly lower scores on five scales, namely, RP, VT, RE, and MH of SF-36, and PCS, demonstrating a reduction in their HRQOL compared with patients without antiviral therapy. The results reflect similar findings from studies comparing HRQOL at baseline and after antiviral treatment in CHC patients with IFN alone or with RBV in addition [11,35,36]. The negative effect of antiviral therapy on RP for CHC patients in this study was 27.7, worse for those without antiviral therapy (ranging from -6.0 to -11.6) [22,23,35], while the negative effect on RE was 34.9 points, also much higher than the impact on CHC patients without antiviral therapy (ranging from -10.1 to -18.1) [22,23,35].

The average CLDQ score of the 108 antiviral-treated patients was 5.23 in this study, indicating a moderate reduction in quality of life among antiviral-treated CHC patients and it is a lower score than that of chronic hepatitis patients treated with IFN-α in Tehran, Iran (5.40) [37], and much lower than norms in the USA (5.9) [38]. Compared to CHC patients without antiviral therapy, antiviral-treated CHC patients demonstrated significantly lower scores on three scales, namely, fatigue, systemic symptoms, and emotional function, in the CLDQ. Reductions in the HRQOL of CHC patients with antiviral therapy were higher in magnitude than that reported for CHC patients without antiviral therapy [20,39,40], indicating that antiviral therapy has a severe impact on CHC patients.

Significant correlations between scores on CLDQ subscales and SF-36 scales were observed. The magnitude of correlations between the fatigue subscale of the CLDQ and SF-36 scales was higher, indicating that fatigue is the most important factor explaining variations in functioning and well-being in CHC patients undergoing antiviral treatment. The highest correlations were between the CLDQ subscale of fatigue and the SF-36 scale of vitality, and the CLDQ subscale of emotional function and the SF-36 mental health scale, which concurred with previous reports [41,42]. This pattern of correlations between the CLDQ and SF-36 subscales provides further evidence for the convergent and discriminant validity of the CLDQ, also indicated by Hauser et al. [43], Ferrer et al. [44], and Younossi [38].

This study showed low PCS and MCS scores, especially during treatment weeks 9–16. The lowest PCS score (35.06) was comparable to one report from France demonstrating progressive decreases in PCS and MCS scores from treatment week 12 (PCS score 36.0, MCS score 41.6) up to month 12 [11]. Similarly, Kang et al. reported the lowest SF-36 scores in all domains at treatment week 12, while scores progressively increased thereafter [35]. This study failed to find a correlation between ALT levels and the patients’ HRQOL, reflecting studies in western countries finding no association between QOL and serum ALT levels [24,34]. Among the demographic variables examined, this study found that a patient’s monthly income was the major predictor of PCS, MCS, and CLDQ scores. This is consistent with previous findings that self-perceived financial stress is the most influential factor [24] and is compatible with research by Bezemer et al. showing correlations between a patient’s income (Euros) and PCS score [16]. This study illustrated that patients with higher incomes have a significantly better QOL during treatment compared to those with lower incomes. While there were no significant differences in other variables except gender on the CLDQ, females experienced a negative impact on their QOL, reflecting findings by Bezemer et al. [16]. This study’s results indicate that a patient’s gender (female), treatment duration (mainly after week 8 and especially during weeks 9–16), and monthly income are predictive factors of the patients’ QOL. This acknowledgement could help healthcare personnel understand a patient’s HRQOL during treatment and guide nurses in providing better management of patient discomfort based on different impacting factors. For example, healthcare providers may offer CHC patients adequate antiviral treatment information before treatment week 8 and help patients apply for financial support from available resources, especially for patients who need extended therapy. In addition, case-management can benefit CHC patients through timely assessment of QOL using both generic and disease-specific questionnaires, which they can consult to determine how to relieve side effects caused by treatment without delay. Furthermore, there is a need to establish diverse programs that can help patients improve their HRQOL through a continuous education curriculum, which also provides nurses with adequate knowledge and skills to achieve best practice in managing patients’ physical and emotional changes during antiviral treatment.

## Conclusion

Our study showed a decrease in the QOL of Taiwanese antiviral-treated CHC patients compared with Taiwanese population norms. The results showed that treatment had a particularly serious negative impact on low income earners, during weeks 9–16 of treatment, and an overall greater effect on female subjects. The findings indicate a need for updated counseling and educational materials designed to provide adequate nursing management and consistent healthcare service to this patient setting. Active assessment and timely healthcare interventions could improve the standard consultation programs as well as patient’s quality of life.

This study was performed at one teaching hospital in central Taiwan; therefore, the results may not be representative of all CHC patients in Taiwan. A limitation of the study was the small sample size, as we only recruited 64 age- and gender-matched CHC patients without antiviral therapy as an additional control group. We are currently performing a longitudinal follow-up study that includes HRQOL measurements and analysis of biochemical parameters for antiviral-treated CHC patients at baseline, during-treatment, and post-treatment. Collaboration with more hospitals and recruitment of more antiviral-treated CHC patients would provide more comprehensive data on patients’ HRQOL. Enhanced awareness and understanding of patients’ needs will ensure better healthcare for antiviral-treated CHC patients in Taiwan.

## Competing interests

No conflict of interest has been declared by the authors.

## Authors’ contributions

SCC, SSY and TCL conceived the study, performed statistical analysis and drafted the manuscript. CCC, CCL and YCC participated in the conception and design of the study. SSY and TCL helped to recruit the patients. SCC contributed to drafting the manuscript. All authors read and approved the final manuscript.
